# 1,3-Bis[(*tert*-butylsulfanyl)methyl]-2,4,6-trimethylbenzene

**DOI:** 10.1107/S1600536813002249

**Published:** 2013-01-31

**Authors:** Evelyn Paz-Morales, Manuel Basauri-Molina, Juan Manuel Germán-Acacio, Reyna Reyes-Martínez, David Morales-Morales

**Affiliations:** a Instituto de Química, Universidad Nacional Autónoma de México, Circuito exterior, Ciudad Universitaria, México, D.F., 04510, México; b Ciencias Básicas e Ingeniería, Recursos de la Tierra, Universidad Autónoma, Metropolitana. Av. Hidalgo Poniente, La Estación Lerma, Lerma de Villada Estado de México, CP 52006, México

## Abstract

The complete mol­ecule of the title compound, C_19_H_32_S_2_, is generated by crystallorgaphic twofold symmetry, with three C atoms lying on the axis. The C_ar_—C—S—C (ar = aromatic) torsion angle is 156.2 (2) °. In the crystal, the mol­ecules are linked by very weak C—H⋯S inter­actions, generating [001] chains.

## Related literature

For pincer complexes, see: Morales-Morales *et al.* (2007 ▶); Morales-Morales (2004 ▶); Serrano-Becerra & Morales-Morales (2009 ▶). For uses of SCS pincer complexes in catalysis, see: Morales-Morales *et al.* (2007 ▶); Singleton (2003 ▶). For the structure of the pincer SCS ligand 1,3-bis­[(naphthalen-2-ylsufan­yl)meth­yl]benzene, see: Padilla-Mata *et al.* (2012 ▶).
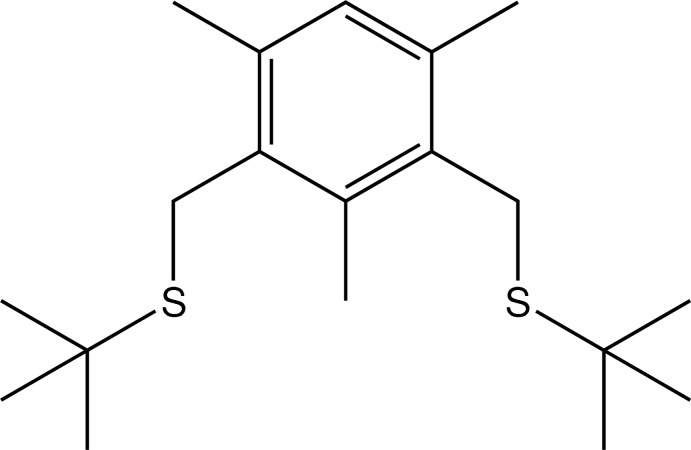


## Experimental

### 

#### Crystal data

C_19_H_32_S_2_
*M* *_r_* = 324.57
Monoclinic, 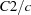

*a* = 14.870 (4) Å
*b* = 14.233 (3) Å
*c* = 9.245 (2) Å
β = 103.693 (4)°
*V* = 1901.1 (8) Å^3^
*Z* = 4
Mo *K*α radiation
μ = 0.27 mm^−1^
*T* = 298 K
0.34 × 0.09 × 0.06 mm

#### Data collection

Bruker SMART APEX CCD diffractometer
10088 measured reflections
1743 independent reflections
1022 reflections with *I* > 2σ(*I*)
*R*_int_ = 0.084

#### Refinement

*R*[*F* ^2^ > 2σ(*F* ^2^)] = 0.055
*wR*(*F* ^2^) = 0.143
*S* = 0.93
1743 reflections
102 parameters
H-atom parameters constrained
Δρ_max_ = 0.30 e Å^−3^
Δρ_min_ = −0.15 e Å^−3^

### 

Data collection: *SMART* (Bruker, 2007 ▶); cell refinement: *SAINT* (Bruker, 2007 ▶); data reduction: *SAINT*; program(s) used to solve structure: *SHELXS97* (Sheldrick, 2008 ▶); program(s) used to refine structure: *SHELXL97* (Sheldrick, 2008 ▶); molecular graphics: *SHELXTL* (Sheldrick, 2008 ▶) and *ORTEP-3 for Windows* (Farrugia, 2012 ▶); software used to prepare material for publication: *SHELXTL* and *PLATON* (Spek, 2009 ▶).

## Supplementary Material

Crystal structure: contains datablock(s) I, global. DOI: 10.1107/S1600536813002249/hb7031sup1.cif

Structure factors: contains datablock(s) I. DOI: 10.1107/S1600536813002249/hb7031Isup2.hkl

Supplementary material file. DOI: 10.1107/S1600536813002249/hb7031Isup3.cml

Additional supplementary materials:  crystallographic information; 3D view; checkCIF report

## Figures and Tables

**Table 1:** Hydrogen-bond geometry (Å, °)

*D*—H⋯*A*	*D*—H	H⋯*A*	*D*⋯*A*	*D*—H⋯*A*
C10—H10*A*⋯S1^i^	0.96	3.11	3.980 (5)	151

Symmetry code: (i) 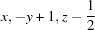.

**Fig. 1.**
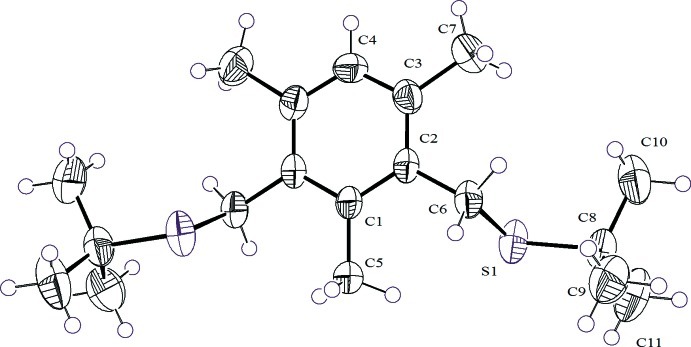
The molecular structure of the title compound with displacement ellipsoids shown at the 40% probability level. Unlabelled atoms are generated by (1-x, y, 1/2-z).

**Fig. 2.**
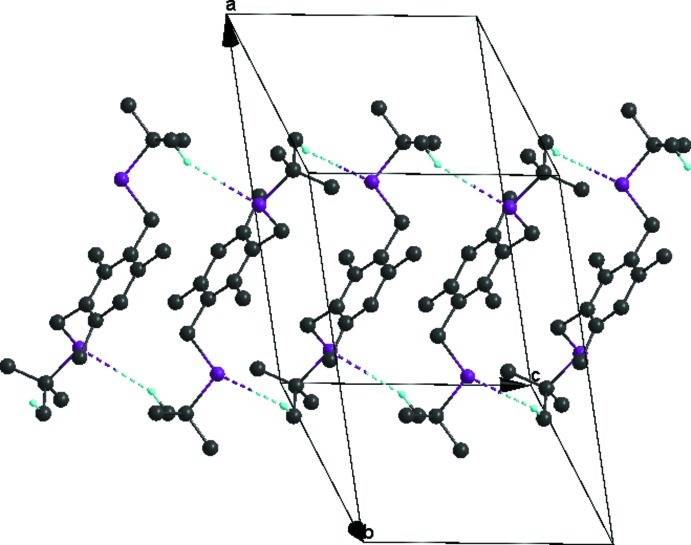
Representation of C—H···S interactions founded in the title compounds. The hydrogen atoms not involved in the hydrogen bonding interactions were omitted.

## supplementary crystallographic information

### Comment

The fine tuning of both steric and electronic properties of pincer ligands is one of the most important goals for both organic and inorganic chemists nowdays, being this possible by the systematic variations and change of both the donor atoms and their substituents in the side arms of this current widely used ligands. Through the years, pincer complexes have become an important tool for synthetic organic chemists, mostly due to their well know robustness, thermal stability and unusual reactivities (Morales-Morales *et al.*, 2007; Morales-Morales, 2004; Serrano-Becerra *et al.*, 2009). Recently, non-phosphine-based ligands and their transition metal complexes have gained considerable attention as suitable and valuable alternatives in transition metal catalysed organic transformations. In this sense, SCS pincer complexes have shown to be efficient as potential catalysts in aldol reactions and Mizoroki-Heck and Suzuki-Miyaura couplings (Singleton, 2003). Previously, we have reported the structure of the pincer SCS ligand 1,3-Bis[(naphthalen-2-ylsufanyl)methyl]benzene (Padilla-Mata *et al.*, 2012). Thus, in this opportunity we report here the crystal structure of the potential pincer ligand [2,4,6-trimethyl-1,3-bis(*tert*-butylsulfanyl)methyl]benzene, the molecular structure is shown in Figure 1.

In the asymmetric unit only half molecule of the title compound is found and a twofold axis is needed to complete the molecule. The (*tert*-butylsulfanyl)methyl moieties are up and down the plane of the phenyl ring with a torsion angle of 156.2 (2)° (C8—S1—C6—C2). The H atoms of the methyl group in the 2 position exhibit disorder in the crystal structure. The molecules in the crystal are linked by weak centrosymmetric intermolecular interactions C10—H10A···S1 with a distance of 3.11 Å, values that are only slightly higher to the sum of the van der Waals radii H—S (3.0 Å). These interactions generate a chain along the *c* axis direction.

### Experimental

To a suspension of NaH (9 mg, 0.38 mmol) on freshly distilled THF (20 ml), 2-methyl-2-propanethiol (30 µ*L*, 0.3 mmol) was slowly added. The resulting reaction mixture was allowed to proceed for 10 min. After this time, a solution of 2,4-bis-bromomethyl-1,3,5-trimethylbenzene (100 mg, 0.3 mmol) in THF (10 ml) was slowly added and the resulting mixture allowed to proceed for further 5 h under stirring at room temperature. After this time the mixture was filtered under vacuum through a short plug of Celite&reg; and the resulting THF solution evaporated in a rotary evaporator to afford the product in a 97% yield. Yellow prisms were obtained by slow evaporation a CH_2_Cl_2_ saturated solution of the title compound.

### Refinement

H atoms were included in calculate positions (C—H = 0.93 Å for aromatic H, C—H = 0.97 Å for methylene H, and C—H = 0.96 Å for methyl H), and refined used riding model, with *U*_iso_(H) = 1.2 *U*_eq_ of the carrier atom. C5 atom is on the twofold axis and their H-atoms were refined with half occupation.

### Crystal data

**Table:**

C_19_H_32_S_2_	*F*(000) = 712
*M**_r_* = 324.57	*D*_x_ = 1.134 Mg m^−^^3^
Monoclinic, *C*2/*c*	Mo *K*α radiation, λ = 0.71073 Å
Hall symbol: -C 2yc	Cell parameters from 2375 reflections
*a* = 14.870 (4) Å	θ = 2.8–24.9°
*b* = 14.233 (3) Å	µ = 0.27 mm^−^^1^
*c* = 9.245 (2) Å	*T* = 298 K
β = 103.693 (4)°	Prism, yellow
*V* = 1901.1 (8) Å^3^	0.34 × 0.09 × 0.06 mm
*Z* = 4	

### Data collection

**Table:**

Bruker SMART APEX CCD diffractometer	1022 reflections with *I* > 2σ(*I*)
Radiation source: fine-focus sealed tube	*R*_int_ = 0.084
Graphite monochromator	θ_max_ = 25.4°, θ_min_ = 2.0°
Detector resolution: 0.83 pixels mm^-1^	*h* = −17→17
ω scans	*k* = −17→17
10088 measured reflections	*l* = −11→11
1743 independent reflections	

### Refinement

**Table:**

Refinement on *F*^2^	Primary atom site location: structure-invariant direct methods
Least-squares matrix: full	Secondary atom site location: difference Fourier map
*R*[*F*^2^ > 2σ(*F*^2^)] = 0.055	Hydrogen site location: inferred from neighbouring sites
*wR*(*F*^2^) = 0.143	H-atom parameters constrained
*S* = 0.93	*w* = 1/[σ^2^(*F*_o_^2^) + (0.0718*P*)^2^] where *P* = (*F*_o_^2^ + 2*F*_c_^2^)/3
1743 reflections	(Δ/σ)_max_ < 0.001
102 parameters	Δρ_max_ = 0.30 e Å^−^^3^
0 restraints	Δρ_min_ = −0.15 e Å^−^^3^

### Special details

**Table:**

Geometry. All e.s.d.'s (except the e.s.d. in the dihedral angle between two l.s. planes) are estimated using the full covariance matrix. The cell e.s.d.'s are taken into account individually in the estimation of e.s.d.'s in distances, angles and torsion angles; correlations between e.s.d.'s in cell parameters are only used when they are defined by crystal symmetry. An approximate (isotropic) treatment of cell e.s.d.'s is used for estimating e.s.d.'s involving l.s. planes.
Refinement. Refinement of *F*^2^ against ALL reflections. The weighted *R*-factor *wR* and goodness of fit *S* are based on *F*^2^, conventional *R*-factors *R* are based on *F*, with *F* set to zero for negative *F*^2^. The thresh*o*ld expression of *F*^2^ > σ(*F*^2^) is used only for calculating *R*-factors(gt) *etc*. and is not relevant to the choice of reflections for refinement. *R*-factors based on *F*^2^ are statistically about twice as large as those based on *F*, and *R*- factors based on ALL data will be even larger.

### Fractional atomic coordinates and isotropic or equivalent isotropic displacement parameters (Å^2^)

**Table:**

	*x*	*y*	*z*	*U*_iso_*/*U*_eq_	Occ. (<1)
S1	0.27026 (6)	0.42315 (7)	0.11668 (9)	0.0588 (3)	
C1	0.5000	0.4725 (3)	0.2500	0.0394 (10)	
C2	0.43183 (18)	0.5219 (2)	0.1470 (3)	0.0394 (7)	
C3	0.4300 (2)	0.6195 (2)	0.1501 (3)	0.0450 (8)	
C4	0.5000	0.6656 (3)	0.2500	0.0493 (12)	
H4	0.5000	0.7309	0.2500	0.059*	
C5	0.5000	0.3672 (3)	0.2500	0.0533 (12)	
H5A	0.4374	0.3447	0.2240	0.064*	0.50
H5B	0.5301	0.3447	0.3473	0.064*	0.50
H5C	0.5325	0.3447	0.1787	0.064*	0.50
C6	0.3595 (2)	0.4693 (2)	0.0319 (3)	0.0457 (8)	
H6A	0.3887	0.4180	−0.0090	0.055*	
H6B	0.3319	0.5113	−0.0489	0.055*	
C7	0.3549 (2)	0.6770 (3)	0.0509 (4)	0.0659 (11)	
H7A	0.3681	0.7426	0.0682	0.079*	
H7B	0.2964	0.6626	0.0727	0.079*	
H7C	0.3523	0.6624	−0.0514	0.079*	
C8	0.1680 (2)	0.4088 (3)	−0.0369 (4)	0.0602 (10)	
C9	0.1911 (3)	0.3565 (3)	−0.1670 (4)	0.0769 (12)	
H9A	0.2187	0.2970	−0.1333	0.092*	
H9B	0.2338	0.3931	−0.2071	0.092*	
H9C	0.1355	0.3465	−0.2428	0.092*	
C10	0.1274 (3)	0.5047 (3)	−0.0864 (5)	0.0895 (14)	
H10A	0.1707	0.5401	−0.1267	0.107*	
H10B	0.1149	0.5375	−0.0026	0.107*	
H10C	0.0709	0.4970	−0.1613	0.107*	
C11	0.1000 (3)	0.3515 (3)	0.0281 (5)	0.0908 (14)	
H11A	0.0433	0.3434	−0.0461	0.109*	
H11B	0.0876	0.3839	0.1124	0.109*	
H11C	0.1263	0.2910	0.0590	0.109*	

### Atomic displacement parameters (Å^2^)

**Table:**

	*U*^11^	*U*^22^	*U*^33^	*U*^12^	*U*^13^	*U*^23^
S1	0.0377 (5)	0.0830 (7)	0.0509 (5)	−0.0087 (5)	0.0007 (4)	0.0007 (5)
C1	0.026 (2)	0.046 (3)	0.047 (3)	0.000	0.0103 (19)	0.000
C2	0.0263 (15)	0.054 (2)	0.0369 (17)	−0.0008 (14)	0.0050 (13)	0.0008 (14)
C3	0.0347 (17)	0.051 (2)	0.049 (2)	0.0049 (15)	0.0097 (15)	0.0073 (16)
C4	0.049 (3)	0.037 (3)	0.063 (3)	0.000	0.014 (2)	0.000
C5	0.036 (2)	0.044 (3)	0.074 (3)	0.000	0.002 (2)	0.000
C6	0.0341 (16)	0.059 (2)	0.0400 (17)	−0.0006 (15)	0.0012 (14)	0.0023 (15)
C7	0.057 (2)	0.067 (2)	0.068 (3)	0.0117 (19)	0.004 (2)	0.0121 (19)
C8	0.0406 (18)	0.071 (2)	0.062 (2)	−0.0081 (18)	−0.0016 (17)	−0.0020 (19)
C9	0.071 (3)	0.086 (3)	0.066 (3)	−0.013 (2)	0.001 (2)	−0.011 (2)
C10	0.055 (2)	0.089 (3)	0.108 (3)	0.012 (2)	−0.015 (2)	0.000 (3)
C11	0.048 (2)	0.118 (4)	0.104 (3)	−0.026 (3)	0.014 (2)	−0.013 (3)

### Geometric parameters (Å, º)

**Table:**

S1—C6	1.816 (3)	C7—H7A	0.9600
S1—C8	1.829 (3)	C7—H7B	0.9600
C1—C2	1.404 (3)	C7—H7C	0.9600
C1—C2^i^	1.404 (3)	C8—C9	1.522 (5)
C1—C5	1.499 (6)	C8—C10	1.518 (5)
C2—C3	1.390 (4)	C8—C11	1.530 (5)
C2—C6	1.520 (4)	C9—H9A	0.9600
C3—C4	1.383 (4)	C9—H9B	0.9600
C3—C7	1.508 (4)	C9—H9C	0.9600
C4—C3^i^	1.383 (4)	C10—H10A	0.9600
C4—H4	0.9300	C10—H10B	0.9600
C5—H5A	0.9600	C10—H10C	0.9600
C5—H5B	0.9600	C11—H11A	0.9600
C5—H5C	0.9600	C11—H11B	0.9600
C6—H6A	0.9700	C11—H11C	0.9600
C6—H6B	0.9700		
			
C6—S1—C8	105.25 (15)	C3—C7—H7C	109.5
C2—C1—C2^i^	119.9 (4)	H7A—C7—H7C	109.5
C2—C1—C5	120.0 (2)	H7B—C7—H7C	109.5
C2^i^—C1—C5	120.0 (2)	C9—C8—C10	110.4 (3)
C3—C2—C1	120.1 (3)	C9—C8—C11	110.1 (3)
C3—C2—C6	119.5 (3)	C10—C8—C11	110.2 (3)
C1—C2—C6	120.4 (3)	C9—C8—S1	111.5 (3)
C4—C3—C2	118.2 (3)	C10—C8—S1	109.4 (2)
C4—C3—C7	118.7 (3)	C11—C8—S1	105.1 (2)
C2—C3—C7	123.1 (3)	C8—C9—H9A	109.5
C3^i^—C4—C3	123.3 (4)	C8—C9—H9B	109.5
C3^i^—C4—H4	118.3	H9A—C9—H9B	109.5
C3—C4—H4	118.3	C8—C9—H9C	109.5
C1—C5—H5A	109.5	H9A—C9—H9C	109.5
C1—C5—H5B	109.5	H9B—C9—H9C	109.5
H5A—C5—H5B	109.5	C8—C10—H10A	109.5
C1—C5—H5C	109.5	C8—C10—H10B	109.5
H5A—C5—H5C	109.5	H10A—C10—H10B	109.5
H5B—C5—H5C	109.5	C8—C10—H10C	109.5
C2—C6—S1	110.1 (2)	H10A—C10—H10C	109.5
C2—C6—H6A	109.6	H10B—C10—H10C	109.5
S1—C6—H6A	109.6	C8—C11—H11A	109.5
C2—C6—H6B	109.6	C8—C11—H11B	109.5
S1—C6—H6B	109.6	H11A—C11—H11B	109.5
H6A—C6—H6B	108.2	C8—C11—H11C	109.5
C3—C7—H7A	109.5	H11A—C11—H11C	109.5
C3—C7—H7B	109.5	H11B—C11—H11C	109.5
H7A—C7—H7B	109.5		
			
C2^i^—C1—C2—C3	−2.1 (2)	C2—C3—C4—C3^i^	−2.0 (2)
C5—C1—C2—C3	177.9 (2)	C7—C3—C4—C3^i^	177.4 (3)
C2^i^—C1—C2—C6	178.1 (3)	C3—C2—C6—S1	−101.5 (3)
C5—C1—C2—C6	−1.9 (3)	C1—C2—C6—S1	78.3 (3)
C1—C2—C3—C4	4.1 (4)	C8—S1—C6—C2	156.2 (2)
C6—C2—C3—C4	−176.1 (2)	C6—S1—C8—C9	49.3 (3)
C1—C2—C3—C7	−175.3 (2)	C6—S1—C8—C10	−73.2 (3)
C6—C2—C3—C7	4.5 (5)	C6—S1—C8—C11	168.5 (3)

Symmetry code: (i) −*x*+1, *y*, −*z*+1/2.

### Hydrogen-bond geometry (Å, º)

**Table:**

*D*—H···*A*	*D*—H	H···*A*	*D*···*A*	*D*—H···*A*
C10—H10*A*···S1^ii^	0.96	3.11	3.980 (5)	151

Symmetry code: (ii) *x*, −*y*+1, *z*−1/2.

## References

[bb1] Bruker (2007). *SAINT* and *SMART* Bruker AXS Inc., Madison, Wisconsin, USA.

[bb2] Farrugia, L. J. (2012). *J. Appl. Cryst.* **45**, 849–854.

[bb3] Morales-Morales, D. (2004). *Rev. Soc. Quim. Mex.* **48**, 338–346.

[bb4] Morales-Morales, D. & Jensen, C. M. (2007). Editors. *The Chemistry of Pincer Compounds* Amsterdam: Elsevier.

[bb5] Padilla-Mata, E., German-Acacio, J. M., García-Eleno, M. A., Reyes-Martínez, R. & Morales-Morales, D. (2012). *Acta Cryst.* E**68**, o1429. 10.1107/S1600536812015280 PMC3344549 22590311

[bb6] Serrano-Becerra, J. M. & Morales-Morales, D. (2009). *Curr. Org. Synth.* **6**, 169–192.

[bb7] Sheldrick, G. M. (2008). *Acta Cryst.* A**64**, 112–122. 10.1107/S0108767307043930 18156677

[bb8] Singleton, J. T. (2003). *Tetrahedron*, **59**, 1837–1857.

[bb9] Spek, A. L. (2009). *Acta Cryst.* D**65**, 148–155. 10.1107/S090744490804362X PMC2631630 19171970

